# CRISPR-SONIC: targeted somatic oncogene knock-in enables rapid in vivo cancer modeling

**DOI:** 10.1186/s13073-019-0627-9

**Published:** 2019-04-16

**Authors:** Haiwei Mou, Deniz M. Ozata, Jordan L. Smith, Ankur Sheel, Suet-Yan Kwan, Soren Hough, Alper Kucukural, Zachary Kennedy, Yueying Cao, Wen Xue

**Affiliations:** 1 RNA Therapeutics Institute, University of Massachusetts Medical School, Worcester, MA 01605 USA; 2 The Wellcome Trust/Cancer Research UK Gurdon Institute and Department of Biochemistry, University of Cambridge, Cambridge, UK; 3 Program in Molecular Medicine, Department of Molecular, Cell and Cancer Biology, and Li Weibo Institute for Rare Diseases Research, University of Massachusetts Medical School, 368 Plantation Street, Worcester, MA 01605 USA

**Keywords:** CRISPR, Liver cancer, Mouse model, Oncogene, RAS

## Abstract

CRISPR/Cas9 has revolutionized cancer mouse models. Although loss-of-function genetics by CRISPR/Cas9 is well-established, generating gain-of-function alleles in somatic cancer models is still challenging because of the low efficiency of gene knock-in. Here we developed CRISPR-based Somatic Oncogene kNock-In for Cancer Modeling (CRISPR-SONIC), a method for rapid in vivo cancer modeling using homology-independent repair to integrate oncogenes at a targeted genomic locus. Using a dual guide RNA strategy, we integrated a plasmid donor in the 3′-UTR of mouse β-actin, allowing co-expression of reporter genes or oncogenes from the β-actin promoter. We showed that knock-in of oncogenic Ras and loss of p53 efficiently induced intrahepatic cholangiocarcinoma in mice. Further, our strategy can generate bioluminescent liver cancer to facilitate tumor imaging. This method simplifies in vivo gain-of-function genetics by facilitating targeted integration of oncogenes.

**Electronic supplementary material:**

The online version of this article (10.1186/s13073-019-0627-9) contains supplementary material, which is available to authorized users.

## Background

Genome editing has been revolutionized by the CRISPR/Cas9 system [1]. CRISPR/Cas9 introduces double-strand breaks (DSBs) at a target genomic locus through an easily programmable single guide RNA and the Cas9 enzyme. CRISPR/Cas9-mediated DSBs typically trigger one of two major pathways for DNA repair: (1) *homology-independent repair*, such as non-homologous end recombination (NHEJ), or (2) *homology-directed repair (HDR)* with an endogenous or exogenous homologous DNA template [1, 2]. Homology-independent pathways, i.e., NHEJ, have been widely used for disruption of genes by inducing either frame shifts or premature stop codons, while HDR is frequently used to precisely edit genes or insert large sequences. However, the utility of HDR for in vivo gene knock-in has been limited by the relatively low editing efficiency of HDR compared to that of the NHEJ pathway [3].

Previously, we and others showed CRISPR/Cas9 could be employed to generate loss-of-function mutations in tumor suppressor genes, making in vivo cancer studies more efficient [4–11]. Gain-of-function alleles can also be made by CRISPR/Cas9 and HDR donor [5, 12, 13]. However, the low efficiency of HDR-based gene knock-in presents a significant hurdle for researchers aiming to model in vivo gain-of-function mutations in oncogenes.

While the overall cancer death rate continues to decline, cancer is still the number two cause of death nationally, in part, due to our limited understanding of the underlying biology of many cancer types. New methods of rapid in vivo modeling are needed to understand cancer biology. Liver cancer is a major cancer type with poor prognosis [14, 15]. Specifically, liver cancer incidence in the USA has tripled in the last 40 years, and liver cancer deaths have been increasing since 2000 [16]. Transposon-mediated integration utilizing sleeping beauty system is an established way to study multiple gain of function of oncogenes in the liver [17–19]. Although transposons are powerful genetic tools, it is difficult to control the integration site and copy number. Thus, a rapid and precise method allowing a greater degree of targeted somatic oncogene knock-in is needed for liver cancer.

Recent studies have demonstrated that homology-independent pathways can be utilized to insert large sequences in vivo [20–22]. Two guide RNAs make knock-in feasible: one guide RNA targets the genomic insertion locus, and the other linearizes the non-homologous plasmid template containing the desired sequence for integration. A similar strategy using 28 bp microhomology arms has also been reported [23].

Here we developed CRISPR-based Somatic Oncogene kNock In for Cancer Modeling (CRISPR-SONIC), a method for rapid in vivo cancer modeling. To first validate our approach, we started with the in vitro integration of a GFP reporter sequence into the 3′-UTR of β-actin. We confirmed this integration by analyzing and sequencing genomic PCR amplicons and by assessing GFP expression driven by the endogenous mouse β-actin promoter through flow cytometry. Next, we used hydrodynamic injection to deliver the CRISPR-SONIC system to somatic hepatocytes and found targeted genomic integration of both GFP reporter and oncogenes such as *HRAS*^*G12V*^ or *Kras*^*G12D*^. Both integrated *HRAS*^*G12V*^ and *Kras*^*G12D*^ are functional in vivo, demonstrated by rapid induction of intrahepatic cholangiocarcinoma (ICC) in mice [24]. Moreover, we further showed our strategy could be used to generate bioluminescent in vivo cancer models.

## Methods

### CRISPR vector

Guide RNAs targeting *Actb* and *p53* were cloned into the pX330 (Addgene 42230) vectors using standard protocols and primers (Additional file 1: Table S1 and S2) [25]. The sgA guide targeting the donor plasmid (Addgene, 83807) and IRES-GFP donor were purchased from Addgene (Addgene, 83575). IRES-HRAS^G12V^ was cloned into Addgene 83575. IRES-Kras^G12D^-IRES-GFP and IRES-Kras^G12D^-IRES-luciferase were Gibson cloned into Addgene 83575.

### Cell culture

The Neuro2a cell line is from the University of Massachusetts Medical School Cell line Freezer program originally sourced from ATCC. The 293 fs, GreenGo, and KP cells are from Dr. Tyler Jacks. Cells were cultured in Dulbecco’s modification of Eagle’s medium (DMEM) (Corning 10-013CV) with 10% serum (vol/vol) and 1% penicillin/streptomycin (vol/vol) under standard cell culture conditions, 37C in 5% CO_2_ tissue culture incubator.

### Transfection

Neuro2A and GreenGo cells were cultured in 6-well plate at 30% confluency for transfection. Lipofectamine 3000 (Invitrogen, L3000015) was used for GreenGo cell transfection, and Lipofectamine 2000 (Invitrogen, 11668027) was used for Neuro2A cell transfection according to the manufacturer’s instructions. A total of 2-μg DNA was transfected per well (i.e., 0.66 μg sgA, 0.66 μg SgActin-Cas9, 0.66 μg Donor). Cells were collected for either flow cytometry or genomic DNA isolation 5 days post-transfection.

### Transduction and infection

The 293 fs cells were plated at 40% confluency in 6-well plates 1 day prior to transduction. The 293 fs cells were transfected for lentiviral production with 600 ng sgActin-3′-UTR_1, sgActin3′-UTR_2, sgNon-targeting, or sgSf3b3 with packaging plasmids [26]. KP cells, Kras^G12D^;p53^−/−^ mouse lung cancer cells [26], were infected with lentivirus (+ 2.5 μg/ml polybrene) and selected with 1.5 μg/ml puromycin.

### Cell viability assay

Two thousand cells post-selection were seeded in black-wall clear bottom 96-well plates in 12 wells per sample. Seventy-two hours after plating, cell viability was assessed using Promega Cell Titer Glo Luminescent Viability Assay (G7570) as per the manufacturer’s instructions. Luminescence at an integration time of 1 s was measured on a plate reader.

### Colony formation

Two thousand cells postselection were seeded in 6-well plates and incubated for 10 days. After 10 days, cells were first fixed with 4% formalin and then stained with 0.5% crystal violet solution. Plates were imaged with a Nikon scanner.

### Immunoblot

Whole cell extracts were lysed in RIPA buffer treated with 1:100 Halt phosphatase cocktail inhibitor (Thermo Fisher 78,420) and 1:50 Roche Complete protease inhibitor (11836145001). Lysates were boiled for 5 min at 95C with Nupage 4X Sample buffer (Invitrogen NP0007). Equal amounts of protein from whole cell extracts were separated on a 4–12% Bis Tris gel and transferred to a nitrocellulose membrane and blocked with Odyssey Blocking buffer. Blots were probed with primary antibodies B-actin 1:1000 (CST 4970), Gapdh 1:1000 (EMD MAB274), Hras 1:500 (Millipore OP23), and GFP 1:2000 (CST 2956) overnight at 4C. Blots were then incubated with a fluorescent secondary antibody (LICOR) and imaged on the Odyssey Imaging Platform.

### Genomic DNA purification and PCR

Genomic DNA was purified from cells using the Roche Genomic DNA purification kit (Cat no. 11796828001) at least 5 days post-transfection. One hundred-nanogram genomic DNA were used as template for sgActin integration PCR, and 300 ng genomic DNA was used as a template for sgp53 integration. LA-Taq (Clontech) or Herculase II (Agilent) was used for PCR.

### TOPO cloning

PCR amplicons were first gel extracted using Qiagen QIAquick Gel Extraction kit (28704) as per the manufacturer’s instructions. Gel-purified PCR fragments were then TOPO cloned using the Zero Blunt TOPO PCR cloning kit (K2835) for sequencing. TOPO clones were miniprepped using the Qiagen Spin Miniprep kit (272014), and Sanger sequencing was performed by Genewiz.

### Flow cytometry

Cells were transfected with lipofectamine 3000 as described above. Five days after transfection, cells were trypsinized, washed, and re-suspended in 500 μL PBS and loaded to flow cytometry (BD, Accuri™ C6) for detection of GFP-positive cells. Twenty thousand events were collected for each sample. Samples were analyzed and gated for dead cells and singlets using Flow Jo Software.

### Hydrodynamic tail vein injection

All plasmids used for the in vivo study were purified by Qiagen Maxi-Prep Endotoxin-free Kit (Qiagen, 12,362) according to the manufacturer’s instructions. Fifteen micrograms per plasmid per mouse was mixed together in 2 ml 0.9% sterile saline at room temperature. Plasmids were then delivered to mice by hydrodynamic tail vein injection. Specifically, within 5–7 s, all 2 ml mixed plasmids were continuously and smoothly injected [19]. Mice were then warmed by heat lamp for 30 min to recover from injection shock.

### Histology and immunohistochemistry

Mouse liver tissue was collected from sacrificed mice, fixed with 4% formalin overnight followed by dehydration for 24+ hours in 70% ethanol. Tissues were then embedded in paraffin by the UMassMed Morphology Core. H&E staining was performed by the Morphology Core according to common methods on 4-μm paraffin sections. Immunohistochemistry staining was performed following standard protocols. Briefly, tumor sections were deparaffinized with xylene and dehydrated with serial ethanol dilutions. Slides were then boiled for 9 min with 1 mM citrate buffer (pH 6.0) for antigen retrieval. Next, 3% hydrogen peroxide was used to inactivate endogenous peroxidase activities for 10 min at room temperature. Tissues were then blocked with 5% normal horse serum from ImmPRESS™ HRP Anti-Rabbit IgG (Peroxidase) Polymer Detection Kit (Vector labs, MP-7401-50) for 1 h at room temperature. Slides were then incubated with primary antibody against Ck19 1:200 (Abcam, 133,496) or GFP 1:200 (CST, 2956) overnight at 4 °C. Secondary HRP anti-rabbit antibody (Vector labs, MP-7401-50) was incubated with sections for 1 h at room temperature, followed by incubation with substrate/chromogen (Fisher Scientific, TA-125-QHDX). Slides were then counterstained with hematoxylin, dehydrated in ethanol, followed by a xylene wash and sealed with a coverslip for long-term storage. H&E or IHC images were captured using a Leica DMi8 microscope. IHC slides were quantified by selection of five random fields per paraffin embedded section, and positive hepatocytes per  20X field counted.

### IVIS imaging

At the indicated time post injection, mice were given 200 μl luciferin (15 mg/ml) intraperitoneally. Signal was allowed to stabilize for 10 min and then loaded into the Perkin Elmer IVIS machine for capture of luminescent signal (1 min exposure).

## Results

### CRISPR-SONIC-mediated homology-independent IRES-GFP integration in mouse cells

**Fig. 1:**
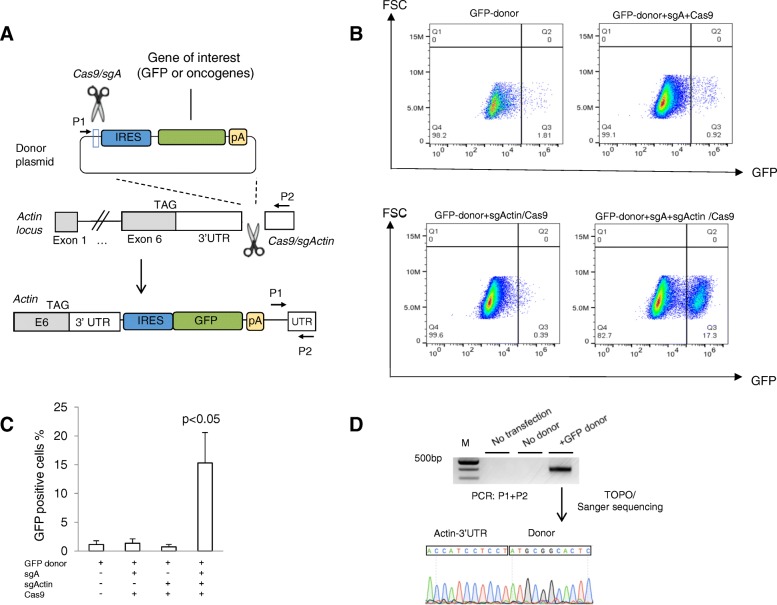
CRISPR-SONIC enables homology-independent IRES-GFP integration in mouse cells. **a** Schematic showing the target genomic locus, guide RNAs, and donor plasmid. **b** Neuro2A cells were transfected with indicated plasmids. Five days post-transfection, cells were trypsinized, washed with PBS, re-suspended in PBS, and analyzed by flow cytometry for detection of GFP-positive cells. **c** Statistical analysis of percentage of GFP-positive cells. Reported as mean percentage. Error bars are s.d. (*n* = 3). **d** PCR detecting genomic integration and representative sanger sequencing. M, molecular weight DNA ladder

Although CRISPR/Cas9 can facilitate the integration of large DNA sequences into a target locus and is applicable for human cells [20–22], a flexible in vivo gene knock-in method has not been established for cancer mouse models. To knock-in desired sequences, we chose the β-actin locus because it is robustly expressed in most mouse cell types and organs [27]. We first designed a guide RNA targeting the 3′-UTR of β-actin locus. We also adopted a published donor plasmid containing IRES (Internal Ribosome Entry Site) sequence to express gene of interest (GOI) and an sgRNA (named sgA) to linearize the circular plasmid donor [21]. We then designed a strategy to flexibly clone any GOI sequence into the donor plasmid (Additional file 1: Figure S1). In this CRISPR-SONIC system, knock-in occurs in three steps: (1) Cas9 and sgActin cuts the 3′-UTR of β-actin; (2) Cas9 and sgA linearizes the donor plasmid; (3) linearized donor is inserted in the 3′-UTR of β-actin through NHEJ. If insertion occurs in the correct orientation, expression of the GOI will be driven by the endogenous β-actin promoter and the IRES signal respectively (Fig. 1a). Finally, the poly A signal ensures transcription termination after the GOI.

We then transfected combinations of two guide RNAs/Cas9 and the plasmid GFP donor into the Neuro2A mouse neuroblastoma cell line and analyzed integration by flow cytometry five days post-transfection (Fig. 1b). FACS analysis detected 15.3 ± 5.3% GFP-positive cells post-transfection (Fig. 1c). Control transfections, GFP donor alone, GFP donor + sgActin/Cas9, and GFP donor + sgA + Cas9 all produced a low percentage level of GFP by flow cytometry 5 days after transfection (Fig. 1b, c). We confirmed successful GFP donor integration in the Neuro2a genome by PCR amplifying the fused GFP donor/β-actin DNA sequence and Sanger sequencing the amplicon (Fig. 1d and Additional file 1: Table S3). To evaluate CRISPR-SONIC efficiency in other cell lines, we performed the same transfection in GreenGo cells and observed low integration efficiency (~ 1%) likely due to a single nucleotide polymorphism (SNP) two nucleotides adjacent to the SgActin PAM sequence reducing CRISPR cutting (Additional file 1: Figure S2).

As described above, CRISPR-SONIC occurs in three steps with sgA linearizing the circular donor plasmid prior to its insertion in the 3′-UTR of β-actin. Next, we tested whether a linear DNA donor could alternatively be used for CRISPR-SONIC. To generate a linear donor, we PCR amplified and gel purified the IRES-GFP donor sequence. We then tested whether our IRES-GFP amplicon could be transfected with sgActin/Cas9 for efficient integration into the 3′-UTR of β-actin. Using Neuro2a cells, we found that approximately 6% of cells were GFP-positive 5 days post-transfection with linear donor, compared to approximately 19% of cells transfected with circular GFP donor, sgA, and Sg-Actin/Cas9 (Additional file 1: Figure S3). Interestingly, co-injection of PCR donor plus sgActin/Cas9 by hydrodynamic injection did not generate GFP-positive cells in mouse liver (data not shown). It is possible that the linear PCR product is not stable in vivo or unable to efficiently translocate into the nucleus. More investigation will be required to understand variances in integration efficiency between circular and linear donor plasmids.

### Efficient and targeted homology-independent integration of IRES GFP in mouse liver

**Fig. 2:**
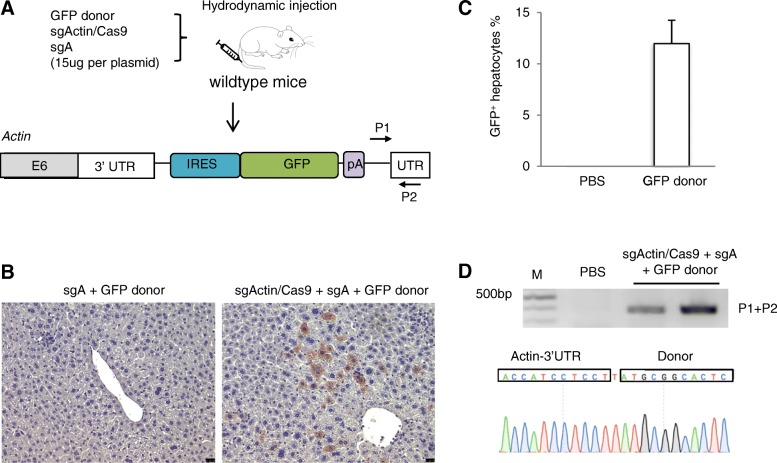
CRISPR-SONIC enables efficient IRES-GFP integration in vivo. **a** Schematic showing the target genomic locus, guide RNAs, and donor plasmid for in vivo integration. Hydrodynamic tail vein injection was performed to deliver the plasmids to FVB mice. Seven days later, mouse livers were collected for histological analysis or genomic DNA purification. **b** IHC was performed to examine the GFP-positive cells from liver slides. Scale bars are 25 μm. **c** Statistical analysis of IHC GFP-positive cells from three mice. Error bars are s.d. (*n* = 1 for PBS and *n* = 3 mice for GFP donor). **d** Genomic PCR detected integration bands. The last two lanes are biological replicate samples

Following in vitro validation of our editing strategy, we then tested our strategy in vivo. We used hydrodynamic tail-vein injection to deliver CRISPR-SONIC to mouse hepatocytes [17–19]. We again used sgActin/Cas9, sgA, and the GFP donor plasmids for targeted integration (Fig. 2a). Seven days after hydrodynamic injection, mice (*n* = 3) were sacrificed for liver tissue collection. By immunohistochemistry (IHC) staining, we detected 12.0 ± 2.3% GFP-positive hepatocytes in vivo (*p* < 0.05 compared to control group) (Fig. 2b, c). Next, we confirmed IRES-GFP sequence integration at the target locus through PCR detection of the expected band and subsequent Sanger sequencing of the amplicon (Fig. 2d).

### Development of intrahepatic cholangiocarcinoma following CRISPR-SONIC of oncogenic *HRAS*^*G12V*^

**Fig. 3:**
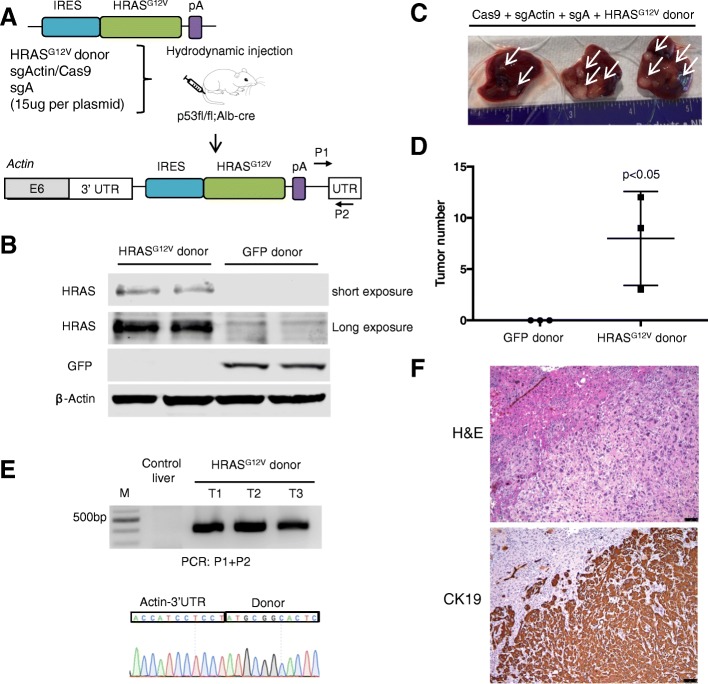
CRISPR-SONIC enables IRES-HRAS^G12V^ integration in vivo for tumor modeling. **a** Schematic showing the target genomic locus, guide RNAs, and donor plasmid for in vivo integration. **b** Western blot confirms HRAS overexpression resulting from targeted integration of Neuro2A cells 5 days post transfection. **c** Liver tumors from injected mice harvested at 1 month. **d** Number of surface liver tumors. Error bars are s.d. (*n* = 3 mice). **e** PCR detected integration band in liver tumor genomic DNA. T1-T3 are tumors from three mice (*n* = 3). **f** Liver tumors are positive for the ICC marker Ck19. Scale bars are 25 μm

ICC is an aggressive cancer type lacking effective therapy [28]. TP53 (26–44% of cases), and KRAS (16–18%) are top driver mutations in ICC [29, 30]. Previous studies showed that oncogenic HRAS or KRAS can cooperate with p53 loss to drive ICC in mouse models using Cre-LoxP or transposons [24, 31]. To determine whether we could apply CRISPR-SONIC to knock-in oncogenic Ras such as *HRAS*^*G12V*^ to model ICC in vivo, we cloned a *HRAS*^*G12V*^ donor plasmid with human HRAS sequence (Fig. 3a) [32]. First, we validated the HRAS donor plasmid in vitro. Five days post-transfection of the CRISPR-SONIC plasmids in Neuro2A cells, we detected HRAS over-expression by western blot using GFP donor as a control (Fig. 3b). Next, hydrodynamic injection was used to deliver the CRISPR-SONIC components to a mouse strain with p53 liver knockout (p53fl/fl; Alb-cre/+) (Fig. 3a) [33, 34]. One month post-injection, we observed gross tumor formation in the livers, 8.0 ± 4.0 tumors per mouse (Fig. 3c, d). As a control, mice injected with sgActin/Cas9, sgA, and GFP donor did not develop liver tumors (Fig. 3d, *p* < 0.05). To confirm that *HRAS*^*G12V*^ was integrated into the β-actin locus, we detected the expected PCR bands and Sanger sequenced the bands (Fig. 3e).

To characterize the genotype at integration sites, we gel purified the integrated amplicons generated by P1,P2 and P3,P4 primer sets and screened 15+ TOPO clones per group (Additional file 1: Figure S4A and B). Using primer set P1-P2 through the Actin-HRAS fused sequence, we did not detect indels in 15/17 clones. In 2 clones, the same complex indel was detected by Sanger sequencing (ATCCTCTTCT) (Additional file 1: Figure S4C and D and Additional files 2 and 3). Using primer set P3-P4 to characterize the Actin-IRES fused sequences, we Sanger sequenced 15 TOPO clones and did not detect any indels (Additional file 1: Figure S4).

Finally, to better characterize the tumors, we collected tumor tissue for histologic analyses. Using IHC, we observed that tumors are positive for the primary ICC marker, cytokeratin 19 (Ck19) (Fig. 3f) [35–38].

### CRISPR-SONIC enables combinatorial Kras^G12D^ knock-in and p53 knockout in immunocompetent mice

**Fig. 4:**
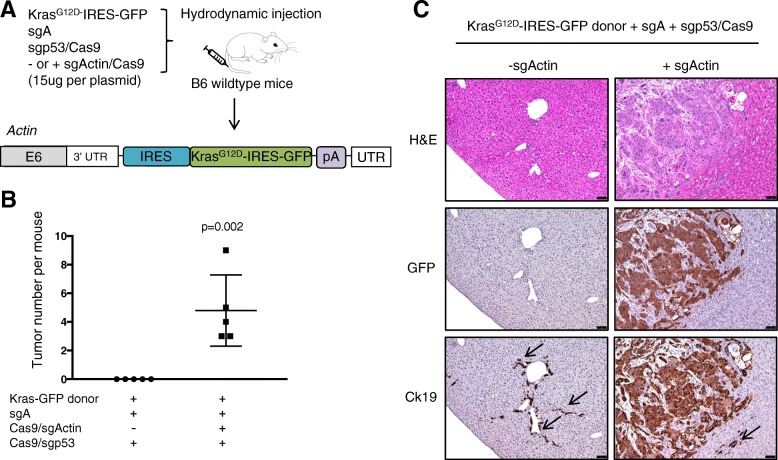
CRISPR-SONIC enables combinatorial Kras knockin and p53 knockout in B6 wildtype mice. **a** Schematic showing the target genomic locus, guide RNAs, and donor plasmid for in vivo integration. **b** Quantification of liver tumor per mouse. Error bars are s.d. (*n* = 5 mice). **c** Representative H&E and IHC staining detected GFP-positive and Ck19-positive tumor cells in +sgActin mice. Arrows denote internal control Ck19 staining in bile ducts. Scale bars are 75 μm

To test whether the use of CRISPR-SONIC could be broadly used to model cancer driven by different oncogenes, we cloned another oncogenic Ras, *Kras*^*G12D*^, into our donor plasmid. ICC driven by Kras^G12D^ also requires loss of function in p53 [24]. To track *Kras*^*G12D*^ integration and expression, we included an IRES-GFP cassette after Kras that would make the resulting tumors GFP positive (Fig. 4a). This dual IRES donor (IRES- Kras^G12D^-IRES-GFP) generated a similar level and percentage of GFP-positive cells compared to the single IRES GFP donor in cells Additional file 1: Figure S5).

Next, we co-injected CRISPR-SONIC plasmids (Kras-donor, sgActin/Cas9, and sgA) with a guide RNA targeting *p53* (sgp53) [6] to knockout p53 in hepatocytes of wildtype B6 mice (termed “+sgActin group” in Fig. 4a). One month post-hydrodynamic injection, we sacrificed this cohort of mice (*n* = 5) and observed liver tumor formation (Fig. 4b). IHC staining revealed GFP-positive and Ck19-positive tumor cells with abundant stroma, a known feature of ICC [35] (Fig. 4c). Concordantly, CRISPR-SONIC also generated liver tumors (*n* = 3) in wildtype FVB mice (Additional file 1: Figure S6).

To characterize the possibility of the donor integrating into and expressing from the sgp53 target site, we performed control hydrodynamic injection using Kras-donor+ sgA+sgp53/Cas9 (termed “-sgActin group” in Fig. 4) (*n* = 5 mice). At 1 month, we did not observe tumor formation, nor did we detect GFP-positive cells by IHC staining (Fig. 4). However, when we performed PCR in liver genomic DNA, we did detect integration of the donor at the p53 locus despite the lack of tumorigenesis or detectable GFP at the assayed time point (Additional file 1: Figure S7). In Neuro2A cells, the *Kras*^*G12D*^-IRES-GFP with Cas9, sgA, and sg*p53* did not generate strong GFP expression (Additional file 1: Figure S5), indicative of limited expression of our donor from the p53 locus. Together, these data suggest that CRISPR-SONIC requires relatively strong endogenous promoters to drive successful expression of the integrated donor or oncogene.

### Generation of bioluminescent tumors using CRISPR-SONIC

**Fig. 5:**
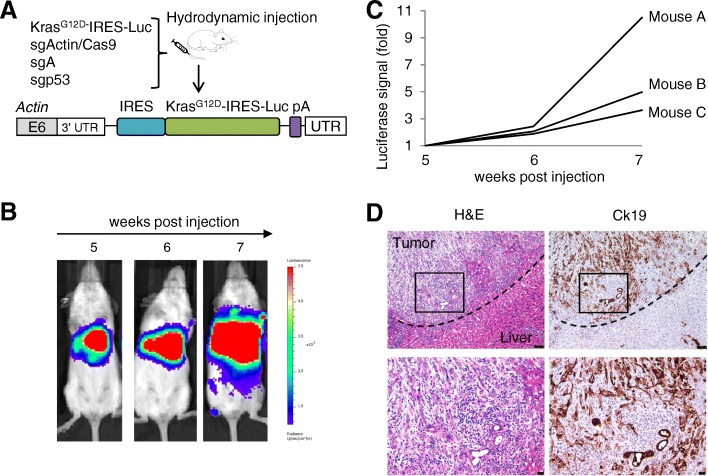
CRISPR/Cas9 enables generation of bioluminescent liver tumors. **a** Schematic showing the target genomic locus, guide RNAs, and donor plasmid. Hydrodynamic tail vein injection was performed to deliver the CRISPR/Cas9 system to FVB mice. **b**, **c** IVIS imaging was performed weekly. **d** H&E and Ck19 IHC staining of liver tumors. Dashed lines denote tumor/liver border. Scale bars are 25 μm

Bioluminescent labeling of tumors enables researchers to monitor tumor initiation and progression in live animals over time. Real-time analysis in live animals provides clear advantages for understanding dynamics of tumor growth. We engineered a donor plasmid containing an IRES-luciferase sequence after oncogenic *Kras*^*G12D*^ and injected mice using the same in vivo integration strategy (Fig. 5a). Five weeks following hydrodynamic injection, we used in vivo bioluminescent imaging to quantify tumor formation over time and observed positive signal indicative of tumorigenesis (Fig. 5b). Weekly imaging allowed for measurement and tracking of tumor growth over time (Fig. 5c). Following live imaging, downstream H&E and IHC staining of Ck19 confirmed ICC formation (Fig. 5d). These data suggest that our method facilitates one-step oncogene and reporter knock-in to facilitate live tumor imaging.

## Discussion

Herein we reported a flexible system for targeted somatic oncogene knock-in to facilitate in vivo cancer modeling. This method enables rapid knock-in of gain-of-function oncogenes and reporter genes in mice.

### CRISPR-SONIC enables efficient homology-independent DNA integration in vivo

Previous somatic integration strategies for cancer modeling, i.e., point mutations or gene knock-in, relied on low-efficiency HDR, limiting the ease of its application both in vitro and in vivo [12, 39]. To satisfy the need for rapid and efficient modeling of cancer in vivo, we turned to CRISPR/Cas9 homology-independent targeted integration. While homology-independent pathways, i.e., NHEJ, are typically more efficient, they are also more error-prone and therefore more frequently utilized for gene disruption rather than DNA integration. To combat this limitation, we adopted a donor with an IRES sequence before the coding region of our gene of interest [21]. Compared to a microhomology-mediated end-joining (MMEJ) strategy to knock in 2A-GFP at Actin [23], small indel mutations before IRES do not affect translation of the gene of interest in our system.

We used the same strategy in vivo and found similarly high efficiency of GFP integration at the target locus. Our IHC staining indicated approximately ~ 10% of the hepatocytes were GFP positive (Fig. 2). This is a significant improvement compared to a 0.5% HDR knock-in efficiency we previously reported using hydrodynamic injection of an HDR donor [5].

### CRISPR-SONIC enables in vivo modeling of ICC

We tested our strategy using Ras isoforms, but presumably any desired oncogenic DNA sequence could be used and tailored to the cancer type of interest. We first chose the well-characterized oncogenic HRAS^G12V^ mutant as a donor and used p53fl/fl;Alb-cre/+ mice which harbor p53 knockout mutations in the liver. As expected, we observed tumor formation after CRISPR-SONIC-mediated *HRAS*^*G12V*^ integration (Fig. 3). We then used an oncogenic *Kras*^*G12D*^ as a donor and delivered this to wildtype B6 or FVB mice along with a guide RNA targeting *p53*. As expected, CRISPR-SONIC delivery of mutant Kras cooperated with sgp53 to induce ICC in vivo (Fig. 4).

Next, we expanded our CRISPR-SONIC system to include knock-in of a bioluminescent marker to enable researchers to follow tumor initiation and maintenance in real time. Integrating this luciferase donor allowed us to dynamically quantify tumor size in our model of ICC (Fig. 5).

### Caveats and solutions

CRISPR-SONIC provides a targeted and higher efficiency knock-in for cancer modeling; however, several caveats need to be considered when applying the technology. First, while CRISPR-SONIC offers a higher degree of genomic targeting than the random genetic insertions associated with transposon delivery, the CRISPR-SONIC system can result in off-target insertions. The donor may insert at off-target sites of the associated sgRNAs or the DSBs that naturally occur in cells [40]. To mitigate the off-target effects of the CRISPR-SONIC system, screening sgRNAs with minimal off-target effects may help to reduce integration at CRISPR off-target sites; although, the donor may still integrate at the DSBs that naturally occur in cells. When the CRISPR-SONIC system (SgA, sgActin, Donor) is co-delivered with a sgRNA targeting a tumor suppressor (i.e., p53) for generation of tumor, CRISPR-SONIC can integrate at both sgRNA sites, sgActin and sgp53. Notably delivery of SgA, sgp53, and donor did not produce either GFP-positive expression in vitro or result in tumor formation in vivo. However, by genomic PCR, we did detect in vivo integration of the donor plasmid at the sgp53 site despite the lack of tumor formation (Fig. 4 and Additional file 1: Figure S7). Future studies are required to determine the degree of off-target donor insertion.

The second major caveat is that the donor vector can be integrated in both orientations [22]. As expected, we also detected inverted integration in the total liver DNA (Additional file 1: Figure S6D). Users should note that CRISPR-SONIC cassettes inserted antisense to the promoter will not be detected by GFP flow cytometry. Further, cassettes inserted distal to a promoter will also go undetected by flow cytometry. During our development of CRISPR-SONIC, we focused on the phenotype driven by the forward orientation of our donor. Future work will determine whether the reverse orientation of the inserted plasmid sequence has adverse effects in vivo. A recently published HITI method used inverted sgRNA sites flanking the GOI to reduce reverse insertion. Reverse insertion without indels will create an intact sgRNA target sequence and will be subjected to additional Cas9 cutting until forward insertion or indels occur to eliminate sgRNA binding sites [22].

Third, the level of expression of the GOI must also be considered when designing sgRNAs and donor plasmids for CRISPR-SONIC. We chose to target the 3′-UTR of the β-actin locus as the preferred target site for integration due to the strong promoter activity of mouse actin. As such, it is possible that the RAS expression level in our system is different from that of transposon RAS [41]. When lower expression is desired, integration can be engineered at the endogenous gene locus [12, 22].

An additional consideration when using the CRISPR-SONIC system is that the Actin 3′-UTR may have a role in regulating β-Actin expression or function. Recent studies showed that Cas9 targeting can induce long deletions which may affect beta-actin function and subsequently cell viability [22, 42]. Of note, infecting KP cells (Kras^G12D^ p53^−/−^ mouse lung cancer cells) with two guides against the actin UTR does not result in reduced expression of β-Actin or notably change cell morphology; however, it does moderately reduce cell proliferation by viability and colony formation assays. The effect is modest compared to an sgRNA targeting an essential gene, Sf3b3. One possible explanation is that the off-target effects of sgActinUTR contributed to the effects on cell viability (Additional file 1: Figure S8). Users should exercise caution when selecting the on-target locus for integration, and further, it is advisable to test multiple guides against the on-target locus before proceeding.

Finally, unlike transposon-based delivery, the CRISPR-SONIC system does not allow for unlimited multiplexing of gain-of-function alleles. Multiple transposons expressing different transgenes can insert in the genome of one hepatocyte [17, 43]. Thus, the transposon delivery system better accommodates users wishing to multiplex 2+ gain of function alleles. However; it should be noted that CRISPR-SONIC could be employed for multiplexing up to two insertions per diploid cell (i.e., two alleles of Actin 3′-UTRs per cell).

## Conclusions

In conclusion, our method facilitates flexible oncogene knock-in to rapidly model cancer in vivo. Further, while we show CRISPR-SONIC delivery via hydrodynamic delivery in the liver, CRISPR-SONIC cancer modeling may also potentially be applied to other tissues with delivery by lentivirus or adeno-associated virus respectively. Finally, CRISPR-SONIC may also be applied to study other genetic diseases including loss-of-function diseases, by knocking-in of a rescue gene at a safe harbor genomic locus.

## Additional files

Additional file 1: **Figure S1.** Cloning strategy to make donor plasmids. Figure S2. CRISPR-SONIC enables IRES-GFP integration in mouse cells. Figure S3. Linear PCR donor generates GFP+ cells in vitro. Figure S4 Sanger sequencing of integration site. Figure S5. Transfection of Kras-IRES-GFP donor with sgp53 is not sufficient to drive GFP expression in cells. Figure S6 CRISPR-SONIC enables combinatorial Kras knockin and p53 knockout in wildtype FVB mice. Figure S7. Kras-IRES-GFP donor can insert at the sgp53 target site. Figure S8. sgActin3′-UTR treatment moderately reduces cell proliferation. Table S1. sgRNA sequences. Table S2. Primer sequences. Table S3. Indels at target integration locus. (PDF 1932 kb)
**Additional file 2:** Sanger sequencing trace with indel. https://figshare.com/s/d76fb6af7195e9761702. (ab1 266 kb)
**Additional file 3:** Sanger sequencing trace with indel. https://figshare.com/s/196e27bf0a7bb9ad8361. (ab1 265 kb)
