# Supplementary material for: CRISPR-SONIC: targeted somatic oncogene knock-in enables rapid in vivo cancer modeling
Source: Genome Med. 2019 Apr 16;11:21. doi: 10.1186/s13073-019-0627-9 (PMC6466773; doi:10.1186/s13073-019-0627-9)
Supplement: Supplementary file 1 — Figure S1. Cloning strategy to make donor plasmids. Figure S2. CRISPR-SONIC enables IRES-GFP integration in mouse cells. Figure S3. Linear PCR donor generates GFP+ cells in vitro. Figure S4 Sanger sequencing of integration site. Figure S5. Transfection of Kras-IRES-GFP donor with sgp53 is not sufficient to drive GFP expression in cells. Figure S6 CRISPR-SONIC enables combinatorial Kras knockin and p53 knockout in wildtype FVB mice. Figure S7. Kras-IRES-GFP donor can insert at the sgp53 target site. Figure S8. sgActin3′-UTR treatment moderately reduces cell proliferation. Table S1. sgRNA sequences. Table S2. Primer sequences. Table S3. Indels at target integration locus. (PDF 1932 kb) Additional file 2: Sanger sequencing trace with indel. https://figshare.com/s/d76fb6af7195e9761702. (ab1 266 kb) Additional file 3: Sanger sequencing trace with indel. https://figshare.com/s/196e27bf0a7bb9ad8361. (ab1 265 kb) [file 13073_2019_627_MOESM1_ESM.pdf]

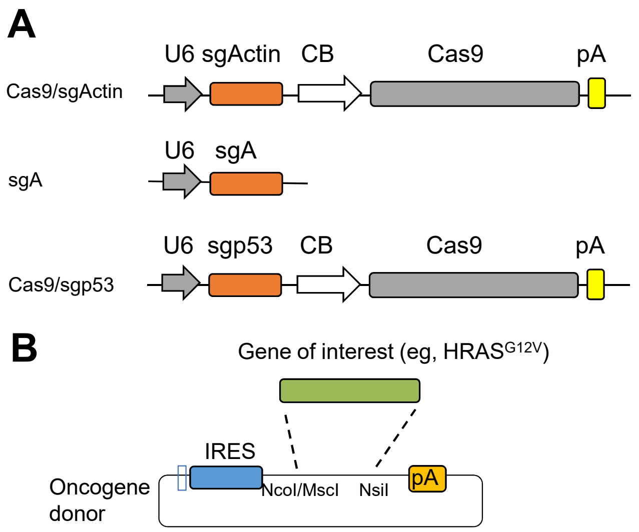

Figure S1 Cloning strategy to make donor plasmids. (**A**) Vector maps. sgActin and sgp53 was cloned into px330 vector as described previously. (**B**) HRAS^{G12V}, Kras^{G12D}-IRES-GFP or Kras^{G12D}-IRES-luciferase was cloned or Gibson-cloned into backbone from GFP donor utilizing restriction enzymes to cut away the GFP sequence.

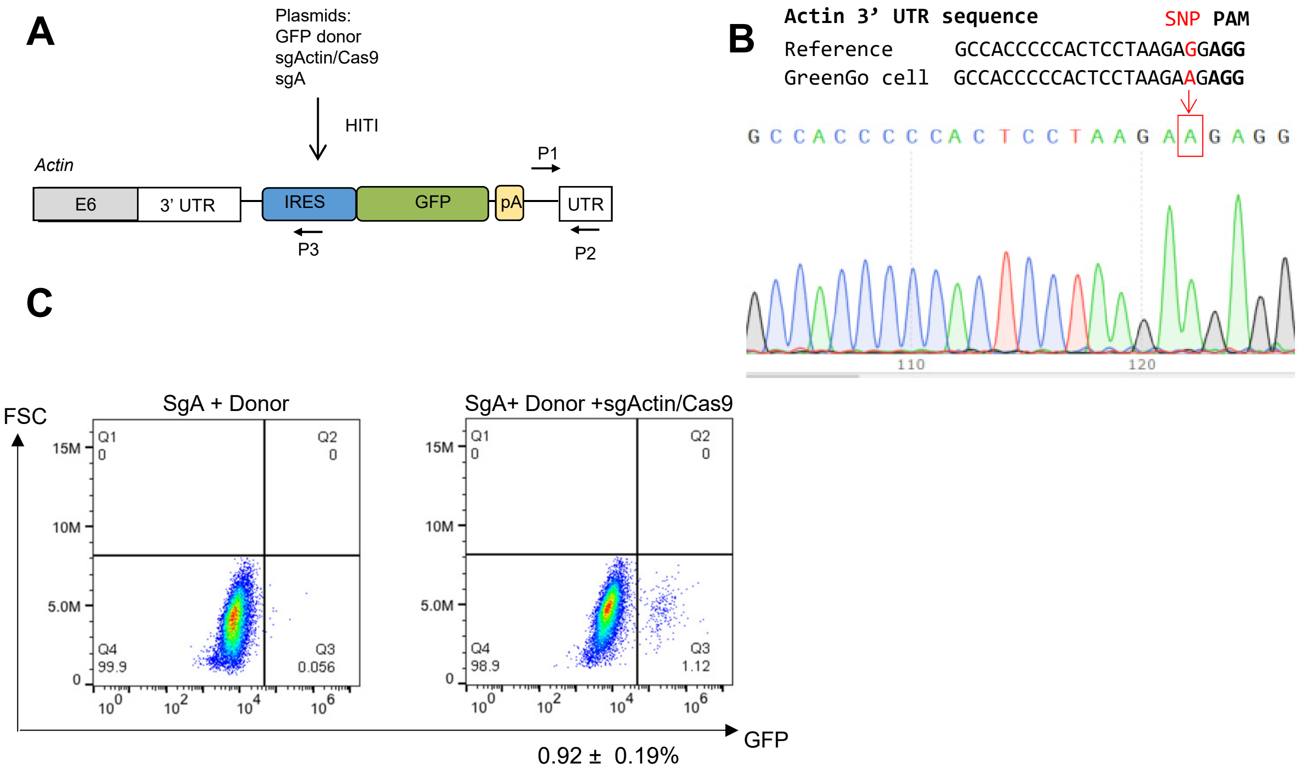

Figure S2 CRISPR-SONIC enables IRES-GFP integration in mouse cells. (**A**) Schematic showing the target genomic locus, guide RNAs, donor plasmid and primers. (**B**) Sanger sequencing showing single nucleotide polymorphism adjacent to SgActin PAM (**C**) Flow Cytometry GreenGo Cells five days post-transfection (n=3).

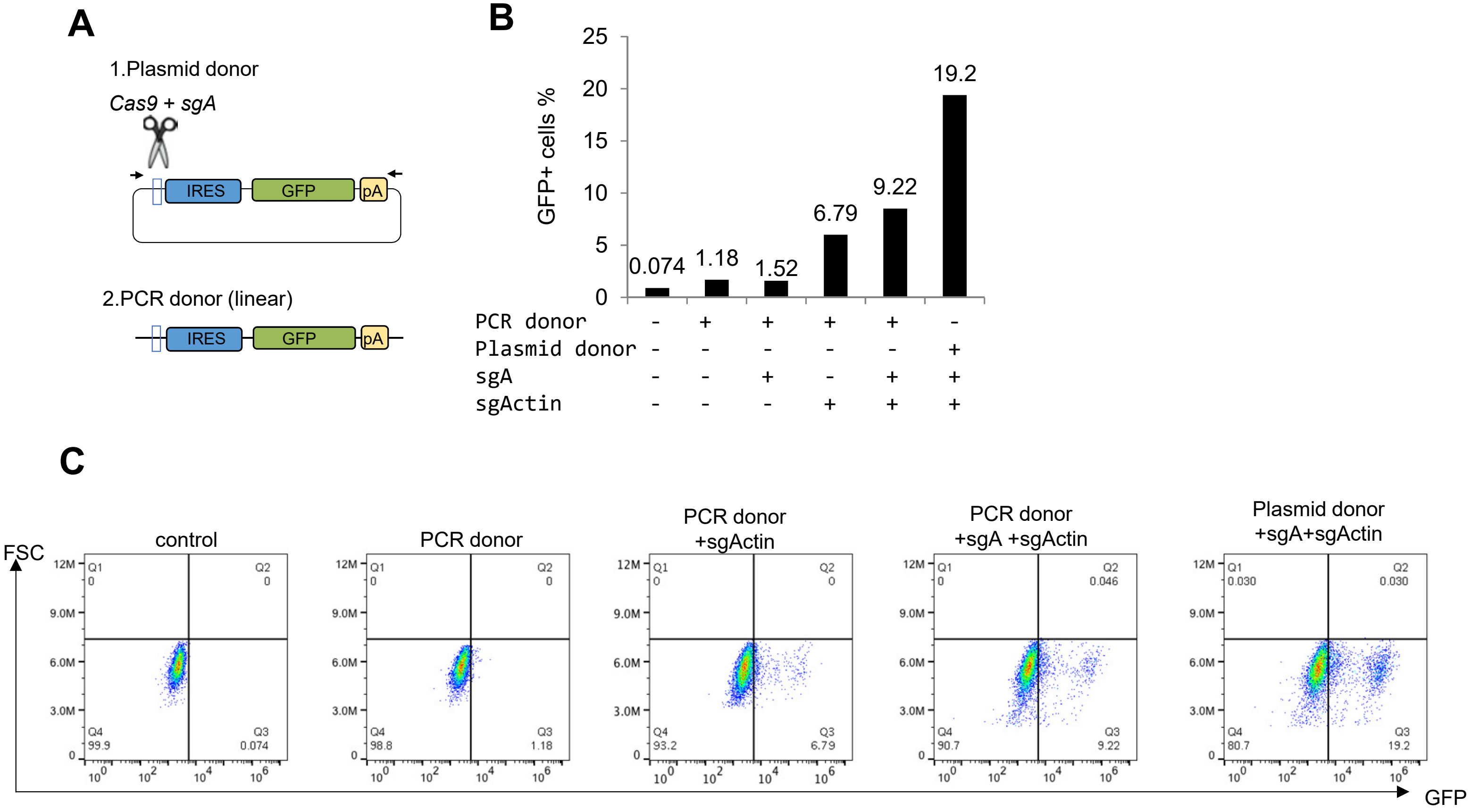

Figure S3 Linear PCR donor generates GFP+ cells *invitro.* (**A**) Schematic of linear and circular donors (**B-C**) Neuro2A cells were transfected with indicated combination. GFP+ cells were quantified by FACS five days post-transfection (n=1).

A

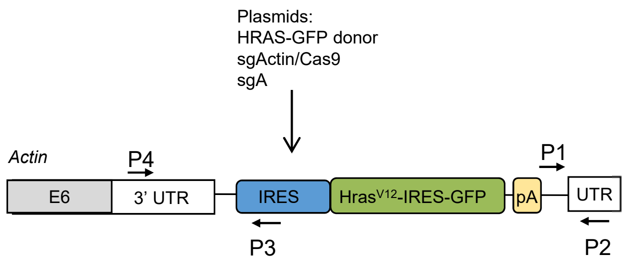

B

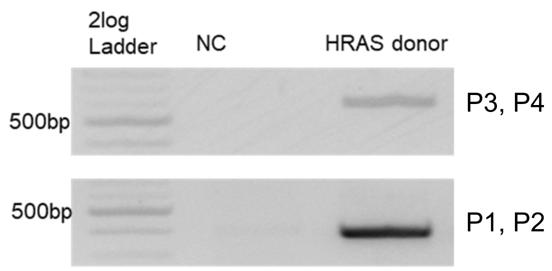

C

Mouse	Fused sequence	Primer Pair	TOPO clones: Indels at fused locus
p53fl/fl;Albcre/+	Actin-IRES	P3-P4	15/15 - No indels
p53fl/fl;Albcre/+	Actin-HRAS	P1-P2	15/17- No indels<br>2/17 – complex indel (ATCCTCTTCT)

D

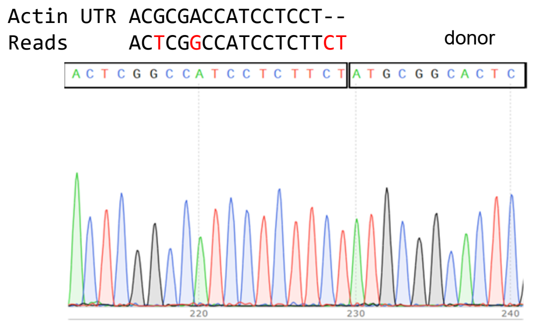

Figure S4 Sanger sequencing of integration site. (**A**) Schematic showing the target genomic locus, guide RNAs and donor plasmid for *in vivo* integration. (**B**) PCR in liver genomic DNA. NC= negative control liver. **(C)** Summary of TOPO cloning sequencing results (n=1). **(D)** Representative complex indel from sanger sequencing, P1-P2 amplicon.

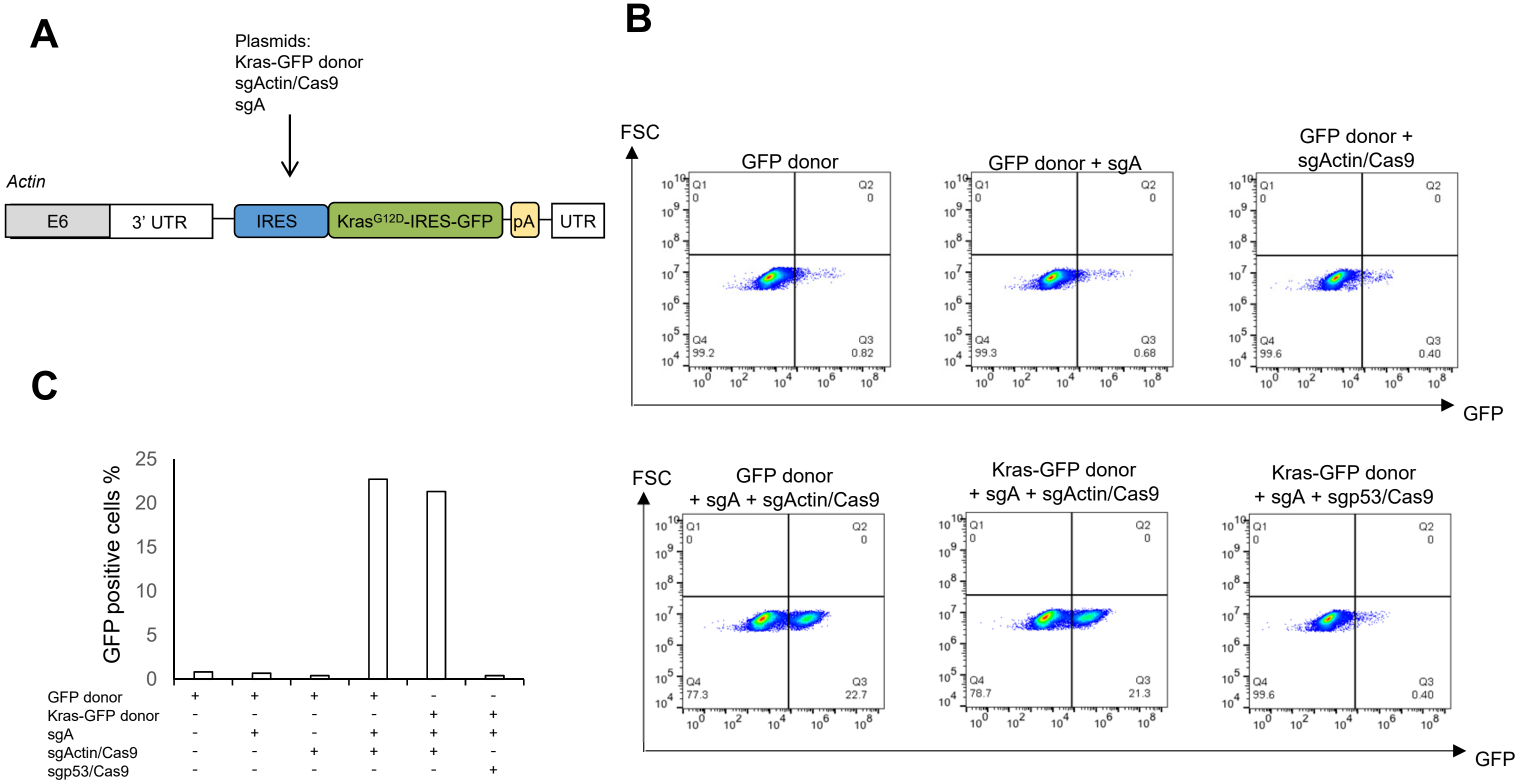

Figure S5 Transfection of Kras-IRES-GFP donor with sgp53 is not sufficient to drive GFP expression in cells. (**A**) Schematic showing the target genomic locus, guide RNAs, and donor plasmid. (**B**) Neuro2A cells were transfected with indicated plasmids. Five days post-transfection, cells were analyzed by flow cytometry for detection of GFP positive cells. (**C**) Quantification of GFP positive cells % from (B) (n=1).

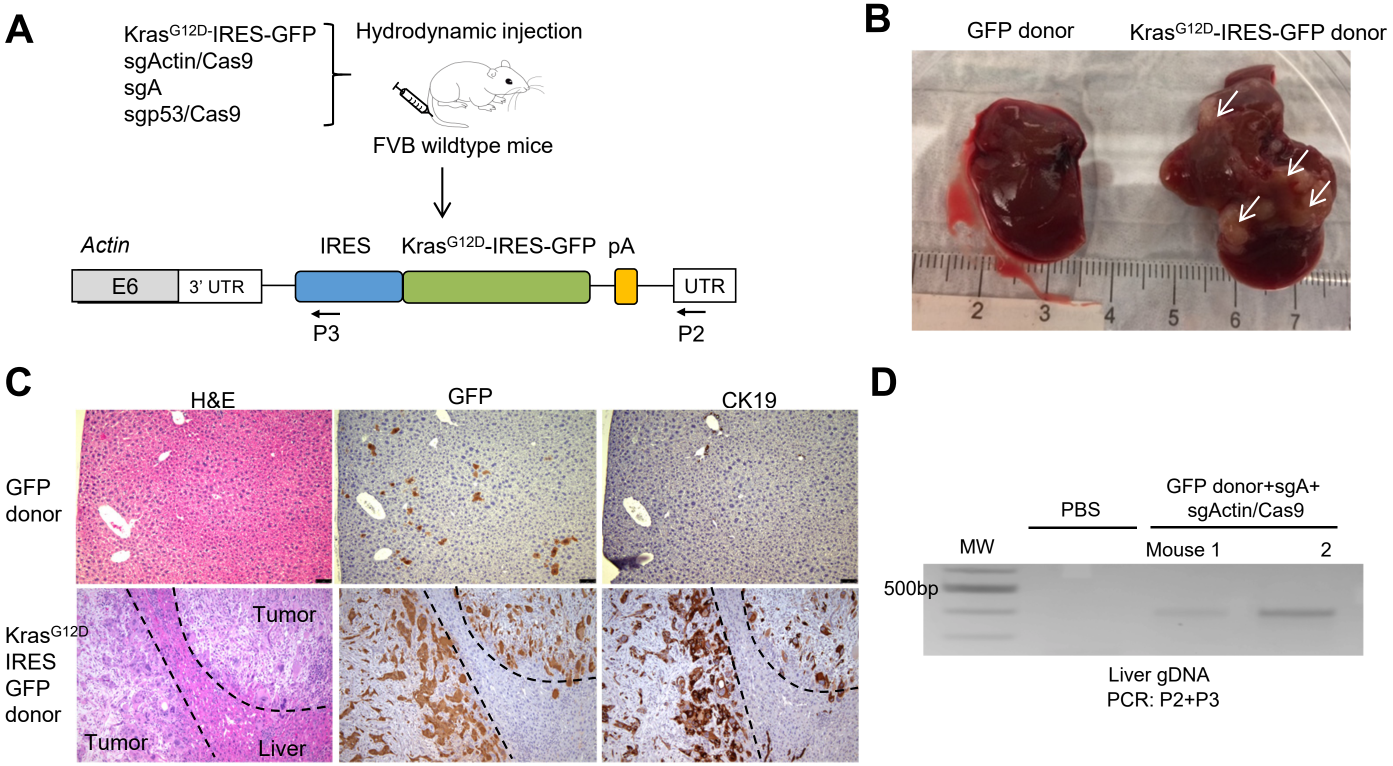

Figure S6 CRISPR-SONIC enables combinatorial Kras knockin and p53 knockout in wildtype FVB mice. (**A**) Schematic showing the target genomic locus, guide RNAs and donor plasmid for *in vivo* integration. (**B**) Representative liver images at 1 month (n=3). **(C)** H&E and IHC staining detected GFP positive and Ck19 positive tumor cells in Kras donor injected mice. Dashed lines denote tumor/liver border. Scale bars are 75 µm. (**D**) Detection of inverted integration *in vivo*. PCR was performed in representative mice using primer P2 and P3 in panel A.

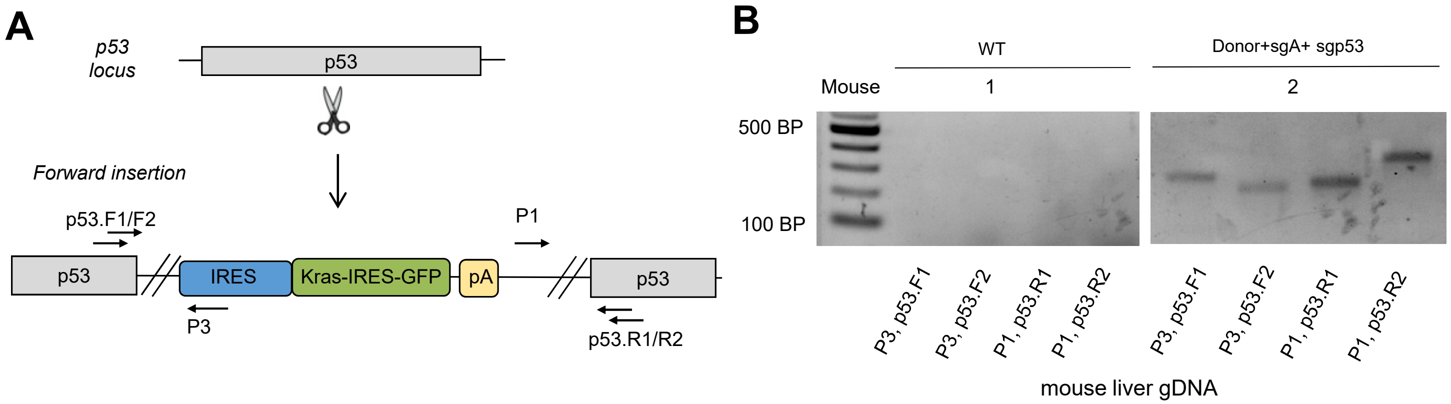

Figure S7 Kras-IRES-GFP donor can insert at the sgp53 target site. (**A**) Schematic showing the target genomic locus, guide RNAs, and donor plasmid. (**B**) PCR in liver genomic DNA. One representative mouse is shown (n=5 mice).

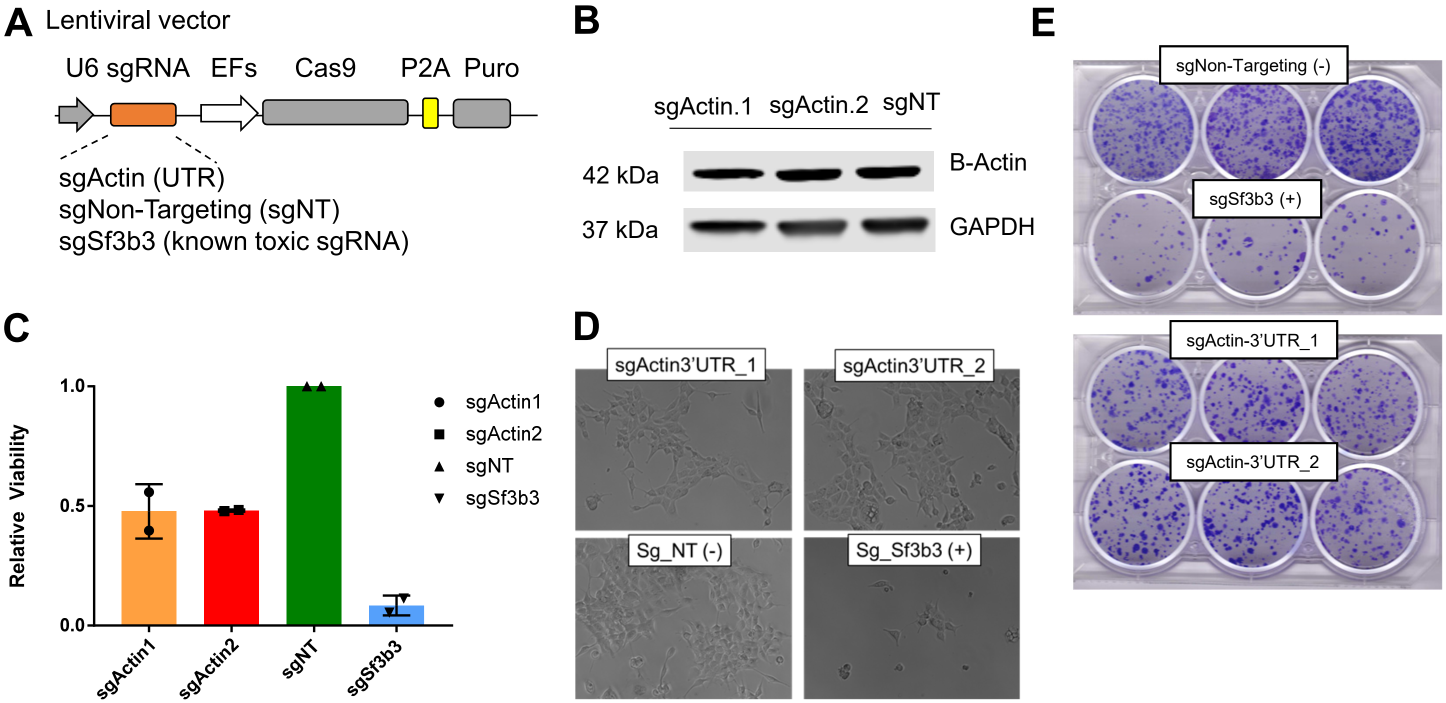

Figure S8 sgActin3'UTR treatment moderately reduces cell proliferation. (**A**) Schematic. $Kras^{G12D};p53^{-/-}$ mouse lung cancer cells were infected with lentivirus and selected with puromycin. (**B**) Representative immunoblot in $Kras^{G12D};p53^{-/-}$ mouse lung cancer cells **(C)** Cell Viability assay at 72 hours, normalized to sgNon-targeting. Error bars are s.d. (n=two biological replicates infections. Each replicate is an average of twelve wells). **(D)** Phase microscopy 10X. **(E)** Colony formation assay at 10 days.

Supplemental Tables

Table S1 sgRNA sequences.

Table S2 Primer sequences.

Table S3 Indels at target integration locus. (indels between actin and donor)

Table S1. sgRNA sequences. An extra "G" is added for U6 transcription.

Gene	Name	Target sequence (5'-3')
	Non-targeting control	GCGAGGTATTCGGCTCCGCG
Actin 3'UTR	sgActin.1	CCACATTTGTAGAACTTTGG
Actin 3'UTR	sgActin.2	GCCACCCCCACTCCTAAGAGG
	sgA	GAGATCGAGTGCCGCATCAC
Trp53	sgp53	GACCCTGTCACCGAGACCCC
Sf3b3	sgSf3b3	GCACAGTATCAAAATACTTG

Table S2. Primer sequences.

Name	Forward primer (5'-3')	Gene to be amplified
P1-1640	CGCCAGGGTTTTCCCAGTCACGAC	Donor plasmid-F
P2-1638	TTGGTCTCAAGTCAGTGTAC	Actin-UTR R
P3-1639	CCTCACATTGCCAAAAGACG	IRES-R
P4-1637	GCTCCTCCTGAGCGCAAGTAC	Actin-UTR F
P53F.1	AGGCTGAGAACACAGTCC	p53
P53F.2	CCATCACCTCACTGCATG	p53
P53_R.1	CGTGCACATAACAGACTTG	p53
P53_R.2	CTAAACTCTGAGGCACAGT	p53

Table S3 Indels at the target integration locus (indels between actin and donor)

Cell or mouse	Fused sequence	TOPO clone Number	Indels at fused locus
Neuro2A cell line	Actin-GFP	3	2 - No indels<br>1 - A deletion
FVB mouse	Actin-GFP	4	3 - No indels<br>1 - T insertion
p53fl/fl;Albcre/+	Actin-HRAS	17	15 – No indels<br>2 – complex indels
p53fl/fl;Albcre/+	Actin-IRES	15	15 - no indels
